# Enhancing Community Participation through Age-Friendly Ecosystems: A Rapid Realist Review

**DOI:** 10.3390/geriatrics8030052

**Published:** 2023-05-11

**Authors:** Judith Sixsmith, Meiko Makita, Deborah Menezes, Marianne Cranwell, Isaac Chau, Mark Smith, Susan Levy, Pat Scrutton, Mei Lan Fang

**Affiliations:** 1 School of Health Sciences, University of Dundee, Dundee DD1 4HJ, Scotland, UK; mmakita002@dundee.ac.uk (M.M.); mcranwell001@dundee.ac.uk (M.C.); i.chau@dundee.ac.uk (I.C.); m.l.fang@dundee.ac.uk (M.L.F.); 2 The Urban Institute, Heriot-Watt University, Edinburgh EH14 4AS, Scotland, UK; debbiemenezes@gmail.com; 3 School of Humanities, Social Sciences and Law, University of Dundee, Dundee DD1 4HN, Scotland, UK; m.z.v.smith@dundee.ac.uk (M.S.); s.levy@dundee.ac.uk (S.L.); 4 Intergenerational National Network, Glasgow G41 1BA, Scotland, UK; patscrutton247@gmail.com

**Keywords:** age-friendly, community participation, ecosystem, older adults, older people

## Abstract

This rapid realist review explored the key components of age-friendly ecosystems that promote community participation among older adults. The study (undertaken in 2021 and updated in 2023) synthesized evidence from 10 peer-reviewed and grey literature databases to identify the underlying mechanisms and contextual factors that shape why, under what circumstances, and for whom an age-friendly ecosystems might be effective as well as the intervention outcomes. A total of 2823 records were initially identified after deduplication. Title and abstract screening produced a potential dataset of 126 articles, reducing to 14 articles after full text screening. Data extraction focused on the contexts, mechanisms, and outcomes of ecosystems for older adults’ community participation. Analysis suggested that age-friendly ecosystems that aim to promote community participation are characterized by the provision of accessible and inclusive physical environments, the availability of supportive social networks and services, and the creation of opportunities for meaningful engagement in community life. The review also highlighted the importance of recognizing the diverse needs and preferences of older adults and involving them in the design and implementation of age-friendly ecosystems. Overall, the study has provided valuable insights into the mechanisms and contextual factors that contribute to the success of age-friendly ecosystems. Ecosystem outcomes were not well discussed in the literature. The analysis has important implications for policy and practice, emphasizing the need to develop interventions that are tailored to the specific needs and contexts of older adults, and that promote community participation as a means of enhancing health, wellbeing, and quality of life in later life.

## 1. Introduction

The demographic shift towards aging societies is a well-recognized global phenomenon, largely due to trends in fertility rates decline and increased longevity due to improved public health and sanitary conditions [1,2]. As of 2022, worldwide there were around 781 million people aged 65 or older, constituting 10% of the world population (1 in 10 people) and projected to double to 1.6 billion by 2050, an increase to 16% (1 in 6 people) globally, with low and middle-income countries experiencing the most rapid increase [2,3]. In the United Kingdom (UK), the population aged 65 and over comprise 11 million of the total population (67.5 million); this age group is experiencing a considerable shift compared to other UK population groups [4]. UK’s baby boomer generation—people currently in their late 50s and 60s—are projected to account for 20% (1 in 5 people) of the total UK population by 2030 when they reach age 65 and over. With increasing life expectancy, within this sociodemographic, the likelihood of experiencing ill health, poverty, loneliness, and isolation is greater than those of the same age in 2002 due to the progression of physical, psychological, social, and financial vulnerabilities as people age [4,5].

This shift in population aging has significant implications for health and social care systems, as well as for individuals, families, and communities. Research shows that aging is not a uniform or linear process, and that biological, psychological, and social factors interact to influence the health and functioning of older adults [6]. While some people may experience disability or chronic illness in later life, others may remain healthy, active, and autonomous well into their 80s and beyond [7]. However, even those who are relatively healthy may face social isolation, financial insecurity, or ageism, which can have negative effects on their quality of life and mental health [2,8]. To address these challenges, it is important to adopt a holistic and person-centered approach to ensure that older people can ‘age in place’, meaning that they are able to live independently and safely for as long as possible in their own homes and communities [9]. This approach recognizes the diversity of older people’s needs and preferences and acknowledges the value of social connections, meaningful activities, and purposeful engagement; it also has prompted a search for effective ways to maintain and improve wellbeing and health-related quality of life as people age [10,11]. To avoid old-age specific silos, intergenerational programs and activities are one promising strategy for enhancing the social integration and support of older people, while also fostering positive attitudes towards aging and intergenerational learning and solidarity—going beyond a focus on the problematization of older people in health and social care terms [12,13].

According to Kaplan, Sanchez, and Hoffman [14], strong intergenerational relationships are not only at the root of healthy and productive aging; they are also an important component of sustainable and livable societies. Intergenerational relationships can take many forms, such as shared housing, mentoring, volunteering, or community service, and can involve individuals, families, or organizations [12,15,16]. This highlights the need for developing social, physical, and technological/digital intergenerational services, spaces, and places that not only accommodate older adults but that welcome them as an integral part of everyday community life. Moreover, research has shown that intergenerational interventions can improve cognitive, emotional, and physical outcomes for both older and younger participants, as well as promote positive attitudes towards aging and reduce ageism and stereotypes [17,18,19,20,21,22].

To ensure that intergenerational programs and services are effective and sustainable, it is important to involve older people and other stakeholders in their design, implementation, and evaluation [23,24,25]. This requires a community-based and participatory approach [26,27] that values the knowledge and expertise of older people and acknowledges their rights and dignity. It also requires a commitment to age-friendly and inclusive environments that support social, physical, and digital accessibility and usability, as well as to integrated and coordinated health and social care systems that recognize the value of prevention, early intervention, and community support [10,13,26,28,29,30].

Building on the traction of the existing age-friendly cities and communities framework [31,32] and Fang and Sixsmith et al.’s [26] work on intergenerational and age-friendly living ecosystems (AFLE), there is a growing recognition that this concept is essential to achieve the United Nations (UN) Sustainable Development Goals 3 and 11. These goals aim to ‘ensure good health and wellbeing for all’ and ‘make cities inclusive, safe, resilient, and sustainable’, respectively [33]. The concept of age-friendly ecosystems is grounded in the belief that older adults should be integrated into their communities and have access to a wide range of opportunities to participate in national and international healthy and active aging initiatives [34]. Nonetheless, this concept developed from the World Health Organization’s (WHO) Age-Friendly Cities and Communities initiative [32], and it is still evolving and being refined [34,35,36]. It has already had a significant impact on how we think about and address the needs of older adults by recognizing they are not just passive recipients of care and support but active participants in their communities and within ecosystems. This approach aims to enable older adults to live well in their later years by fostering community engagement and support. In this context, the current study aims to explore the existing literature to identify how age-friendly ecosystems have emerged and developed, as well as identify the key factors that support effective community participation among older adults to enhance their health and wellbeing.

## 2. Methods

A Rapid Realist Review (RRR) [37,38,39] was undertaken over a 5-month period in 2021 (and updated in 2023) with the aim to identify the contexts, mechanisms, and outcomes of effective community participation of older people in systems, or networks, of interlinked provision. An RRR is a form of evidence synthesis that aims to provide an overview of what works, for whom, under what circumstances, and why in a specific context [38]. The methodological approach draws upon the principles of realist review, a theory-driven approach to systematic review, to explore the underlying mechanisms that influence the outcomes of complex interventions. RRR is characterized by its iterative pragmatic and rapid nature, and has been increasingly used in health and social care research making it a valuable tool to generate relevant and timely evidence for policy and practice decision-making [37,38]. Compared to traditional systematic reviews, RRR is more flexible and allows for the inclusion of a broader range of studies, including grey literature, which can be especially useful for generating evidence in emerging areas of research [38,39,40]. The RRR process consists of six main stages: (1) clarifying the scope of the review (i.e., identifying a review question), (2) identifying relevant studies, (3) extracting and synthesizing the data, (4) engaging stakeholders, (5) validating results, and (6) disseminating results [38].

This review was conducted in a systematic way, and both methods and results are reported according to the Preferred Reporting Items for Systematic Reviews and Meta-Analyses Extension for Scoping Reviews (PRISMA-ScR) checklist (see Table S1).

### 2.1. Identifying the Research Question

In this study, an RRR was conducted to systematically search and synthesize existing knowledge on the application of an ecosystem approach to promoting the community participation of older people. The review question was formulated as follows: How can age-friendly ecosystems support the community participation of older adults?

### 2.2. Theoretical Framework Guiding the Review Process

**Figure 1:**
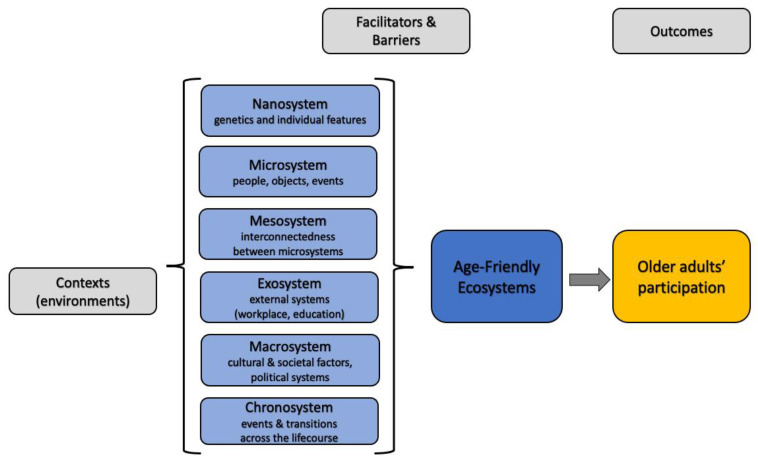
Theoretical framework guiding the RRR. Source: Own elaboration—adapted from Alam’s [43].

After refining the research question, a theoretical framework was created to guide the analytical elements of the review process. The theoretical framework selected, and later adapted, was Bronfenbrenner’s [41,42] ‘socio-ecological systems’ framework. Our own adaptation of this framework was useful for developing the data extraction tool (Section 2.3.4) and conducting data analysis (Section 2.3.5). Bronfenbrenner sought to conceptualize how individuals are both affected by, but also affect, their environment at different nested levels from the nano- and micro- to the exo- and macrosystems. The nano-system comprises the genetics and personal characteristics of the individual (this was not used in the current review since the issue of genetics was not relevant to the review), while the microsystem includes the immediate environment in which they interact. The mesosystem connects these structures, and the exosystem encompasses agencies within the wider social system that affect opportunities for social and civic participation. The macrosystem comprises societal elements that shape culture, values, policies, and laws, and the chronosystem accounts for transitions within the system, especially within the temporal dimension. Together, these levels offer a comprehensive understanding of the systems that influence individuals in their personal and social contexts [26]. As Bronfenbrenner’s framework is grounded on the notion that the individual person, their relationships, local communities, and organizations (e.g., health and social care, voluntary and community groups, leisure, retail, and private and public businesses) are interconnected and shaped by wider cultural influences, it provides a useful framework for locating and contextualizing aging-in-place literature. More specifically, it allows for the identification of interrelated contexts, facilitators, and barriers that impact aging-in-place outcomes, particularly in terms of older adults’ participation in their communities (see Figure 1).

### 2.3. Identifying Relevant Studies

#### 2.3.1. Eligibility Criteria

To ensure the inclusion of relevant and recent literature, specific inclusion and exclusion criteria were employed during the screening and review process. Firstly, only English language studies were considered to prevent potential translation errors and facilitate comprehension. Secondly, the review focused on studies published within the past 10 years to ensure currency of knowledge and practices. Thirdly, the review included studies that addressed all four key concepts of older adults, ecosystem, community, and participation to provide a comprehensive and integrated understanding of these concepts. Finally, all study designs, including opinion pieces and the conclusions of previous literature reviews, were considered to ensure a broad range of evidence was examined while avoiding double-counting of empirical studies.

On the other hand, exclusion criteria were used to exclude studies that were not relevant to the review. Non-English studies were excluded to ensure comprehensibility, and studies published before 2011 were also excluded to maintain a focus on current knowledge and practices. Additionally, studies that did not address all four key review concepts were excluded to ensure comprehensive and integrated insights into these concepts were considered. Adhering to these criteria helped to ensure the identification of valuable and relevant insights into ecosystems for the community participation of older people.

#### 2.3.2. Search Strategies and Databases

The current RRR involved an academic and grey literature review process together with a stakeholder consultation to ground the analysis in academic, policy, and practice contexts, ensuring that the work would produce useful knowledge for time-sensitive, emergent issues [38]. The following 10 databases were searched in 2021 to identify relevant studies reflecting gerontological, social science, health, and social care knowledge: Ageline, Applied Social Sciences Index & Abstracts (ASSIA), Cumulative Index to Nursing and Allied Health Literature (CINAHL+), Google Scholar, Scopus, Social Care Online, PsycINFO, Open Grey, Cochrane Reviews, and Web of Science. Databases were searched for the period 2011–2021. Searches were limited to English language with full-text availability.

The search strategy captured a comprehensive range of studies that investigated the four key concepts of “older people”, “ecosystems”, and “community” and “participation”. To achieve this, a combination of free-text and indexing terms were used. Search terms were initially derived from these four concepts and modified as necessary using Boolean operators (AND/OR) and truncation (e.g., old*). The search strategy was designed to be as inclusive as possible, to ensure that all relevant studies were captured (see Table 1 for search terms and an example of a search string). Additionally, hand searching of the reference lists of retrieved studies was conducted to identify studies that may not have been captured by the initial search. By combining a rigorous search strategy with hand searching, this study was able to conduct a comprehensive search of studies that were relevant to the three key concepts, ensuring that the analysis was grounded in available evidence. This process resulted in the identification of 2852 records.

#### 2.3.3. Screening and Study Selection

The initial 2852 records were subject to a de-duplication process to identify and remove any duplicate records that may have been retrieved from multiple sources. leaving 2823 records. This process helped to ensure that each study was only counted once during the initial screening.

The screening and study selection process for this review was a collaborative effort among the research team. The team began by discussing and agreeing upon the inclusion and exclusion criteria for the review. The main researcher (DM) screened all titles and abstracts (Title/Abs). Other team members then each independently screened 10% of title and abstracts, applying the inclusion and exclusion criteria to identify potentially relevant studies. This meant that two reviewers screened every article title and abstract. Any discrepancies or uncertainties in study selection decisions were discussed among the team members, and a consensus reached. The team also regularly checked in with each other in weekly meetings to ensure that the inclusion and exclusion criteria were being applied consistently. This process resulted in the selection of 126 potentially relevant articles, followed by a full-text screening which involved a thorough examination of each study’s methods, results, and conclusions. After this screening process, 14 studies were selected for inclusion in the final pool (designated * in references section). As with the Title/Abs screening, to ensure the reliability and validity of the results, this last process also involved secondary independent screening of a subset of the selected studies; any discrepancies were discussed and resolved through consensus. By adopting a collaborative approach to study selection, this review was able to draw on the diverse perspectives and expertise of the team members, while also ensuring that the final selection of studies was based on a rigorous and consistent application of the inclusion and exclusion criteria (see Figure S1).

#### 2.3.4. Data Extraction and Charting

To extract relevant data from the selected studies, a data extraction chart was designed and piloted by three reviewers (JS, DM, MF) specifically for this RRR’s approach and research question. The chart included methodology details and basic study characteristics. One reviewer (DM) independently extracted and charted the data from all included studies. At the outset of the data extraction process, a subset of 10% of the full texts were allocated equally across the whole team (co-authors of the paper) for data extraction, and any differences in data extracted were discussed across the team until a consensus was agreed. Only two articles were subject to discussion. The following data, where available, were extracted: author(s), title, publication date, plain summary, DOI, publication type, geographical location of study, study setting, and methodological approach. The charting also included review-specific sections to identify the context, mechanisms, barriers, facilitators, and outcomes related to the application of an ecosystem approach to promoting the community participation of older people.

#### 2.3.5. Data Analysis and Synthesis

Once the data had been charted and verified, they were analyzed using descriptive statistics to identify key patterns and trends in the study characteristics. Additionally, thematic analysis [44] was conducted with the extracted qualitative data concerning contexts, mechanisms, and outcomes. Together, three members of the team analyzed contexts, three members analyzed mechanisms, and three members analyzed outcomes. After undertaking independent open coding, each of the three-member subgroups met regularly to compare coding similarities and discrepancies in terms of the development and naming of themes, as well as the selection of data extracts. By working collaboratively, the subgroups were able to construct and review potential themes together, which were then reviewed and refined by the whole research team in weekly meetings to ensure consistency of analysis and interpretation as well as reflexively identifying how such interpretations had been arrived at. In line with reflexive thematic analysis [44,45], this constant reflexive dialogue helped to ensure the transparency and trustworthiness of the analysis. Any differences that arose during this process were identified, discussed, and resolved through consensus, thereby strengthening the overall conclusions of the review. The data are presented in a narrative format to address the review aim and question.

For this updated version, a new search was conducted on March 12th and 13th, 2023, which involved the same methodology and a thorough search of the original 10 databases for the period from 1 June 2021 to 2023, using the same search strategy as the previous search. Despite the rigorous search effort, only 87 items were retrieved. After title and abstract screening, 85 items were excluded. One article was excluded after full text screening, and the remaining one, authored by Fang et al. [26], raised concerns about potential bias and was ultimately excluded to ensure that the review is conducted in an objective manner. The lack of new publications highlights the need for continued research efforts to address gaps in knowledge and develop new insights (see Figure S2).

### 2.4. Stakeholder Event

As part of the RRR process, a stakeholder event was organized in July 2021 to facilitate a discussion around the review findings. The event was conducted online due to the impact of COVID-19 restrictions on public in-person events. Following a presentation of the project aims and analysis, a diverse group of stakeholders (community members, policymakers, academics, and health and social care practitioners) engaged in discussions around the potential of the ecosystem approach in policy and practice contexts. The insights and feedback gathered from stakeholders during the event were discussed by the research team, comparing them to the initial review findings to establish how the academic knowledge cohered with knowledge in practice contexts. This helped us to gain confidence that the review findings were relevant within a practice and policy context and to identify areas where further collaboration may be needed. This feedback was then used to elaborate a set of recommendations for the success of age-friendly ecosystems (the recommendations are presented in the Discussion section).

## 3. The Analysis

The studies included in this review were sourced from a diverse range of countries, reflecting the global nature of the issue under investigation. The studies were sourced from Brazil [46,47], Canada [48], Iran [49], Ireland [50], Korea [51], Netherlands [52], Portugal [53], and the UK [54]. Most studies derived from the USA [35,36,55,56,57], which may reflect the fact that the issue is particularly prevalent in this region. This diversity helped to ensure that the analysis was not overly biased towards one country context and is instead representative of the global nature of the issue. All studies included were peer-reviewed articles, with one editorial commentary also included [36], and were published between 2012 and 2020 (see Table 2).

### 3.1. Context—What Is an Ecosystem and How Does It Function?

Drawing on the data from the selected sources, ecosystems were defined in a variety of ways using terms such as model, framework, or approach [49,53,56,57]. For example, the Portland and Multnomah County age-friendly initiatives offer a useful way to explore the connection between the World Health Organization (WHO) age-friendly cities framework and the ecological perspective applied to research and action. This approach is being used in a set of age-friendly initiatives co-coordinated by the initiatives’ Advisory Council [57]. Similarly, the AAL4ALL project has created a conceptual architecture that supports an ecosystem of integrated care and assistance services [53]. This conceptual architecture takes a holistic socio-technical approach and reflects on the notion of an ecosystem. Overall, ecosystem definitions were often given in terms of frameworks or approaches that draw on Bronfenbrenner’s work [41,42] or on the Ecology Theory of Aging developed by Lawton and Nahemow [58]. The Ecology Theory of Aging is based on the idea that aging is a process that involves a complex interplay between the individual and their environment. The theory proposes that there are several environmental factors that can influence an individual’s ability to function effectively, including the physical environment (e.g., design and layout of a living space), the social environment (e.g., availability of social support), and the psychological environment (e.g., an individual’s perception of their environment). Bronfenbrenner’s ecological systems framework [42] offers a comprehensive framework for understanding how an individual’s development is shaped by their environment. At the core of this model is the idea that development is not an isolated process but is influenced by multiple environmental systems that interact with each other.

Ecosystems were defined as complex systems consisting of multiple actors, organizations, environments, and interconnections between them. Diverse agents were identified as contributors or actors within ecosystems, including older people themselves as stakeholders, health and care service providers and practitioners, community champions, formal and informal carers, as well as those working within private, voluntary, and community sectors. In all selected studies, except for one [55], ‘older adults’ or ‘older people’ were classified as a homogenous group, primarily disadvantaged by age. This highlights the need for greater consideration of diversity (age, gender, ethnicity, health, and functionality ability) among older people within the context of ecosystems framework, as this is crucial for promoting equity and ensuring that the needs and perspectives of all groups are adequately addressed. Our observation is in line with research [59,60] that acknowledges the importance of diversity and variability in the aging process and population. Despite the need for this, current social gerontology research practices have largely remained consistent with those used in the 1980s and often fail short of applying an intra-age heterogeneity approach, which otherwise would help strengthen the design of policies and programs that benefit people of all ages [59], abilities, experiences, and characteristics.

Within some included sources, ecosystems were identified within a range of environments, including virtual ecosystems such as telecentres in Brazil [47]; local geographically based ecosystems such as pandemic-related initiatives [53]; and ecosystems that inhabit both virtual and geographical spaces [46,49,52]. The selected sources revealed several domains of interest for supporting community participation of older people, including access to care [46,47,50,53], digital inclusion [47,52,54], counseling [56], and maintaining social and physical independence [49,51,52,55,56]. These findings suggest that supporting older adults’ community participation requires a multifaceted approach that addresses their diverse needs and concerns, including access to digital resources, healthcare and counseling services, and opportunities to maintain independence.

Ecosystems were perceived as mechanisms or interventions designed to overcome age-related silos [35,48,54] and transcend disciplinary and sectoral boundaries. These ecosystems were developed to provide more holistic solutions to complex problems [35,46,47,50,52,53,57] and promote collaborative working across professional, academic, and experiential groups [35,36,48,54,57]. Some included sources presented ecosystems as a *service-oriented* system focused on the individual. Service provision-based ecosystems were evident in sources that had government funded provision and healthcare as key focus [46,47,54,57]. Digital organizations were seen as key partners for both communication and organization of services [35,50,53,54]. According to Baldissera et al. [46] an ‘[older adult] care ecosystem’ refers to a system that facilitates the development, organization, and evaluation of virtual organizations to meet the customers’ needs. This definition emphasizes the importance of a coordinated approach that leverages the strengths of diverse partners to create a responsive and effective ecosystem for supporting the needs of older adults.

Some included sources took a community-based approach whereby “engagement in something beyond oneself and for the greater good […] is a form of self-transcendence and most effectively grows out of mindfulness at the individual level” [51] p. 127, which is then supported by services. Four sources [35,36,55,57] argue that communities can become motivated to engage in various age-friendly activities, and that such engagement can help different dimensions of the ecosystem to connect and further support the community. In conceptualizing ecosystems as an intervention, many of these approaches do not necessarily convey the descriptive and often organic nature of Bronfenbrenner’s work. There may certainly be scope to use an understanding of the features of ecosystems to enhance or mitigate certain environmental determinants of wellbeing, but the complex interconnections across systems make planning ecosystems less than straightforward.

A key defining feature of ecosystems revolved around the notion of interconnectedness, more specifically health connectivity [46,50,53] and social connectivity [47,48,54], along with the interconnectedness of the two—for instance, through the social determinants of health [49,51]. Interconnectedness was presented as a means to achieve more holistic and ecological approaches to conceptualizing communities and environments that facilitate wellbeing for older populations [35,36,48,54]. The central relevance of interconnectedness is reiterated by Baldissera et al.’s notion that “collaborative networks for [older adult] care suggest the integration of services from multiple providers, encouraging collaboration to provide better personalized services” [46] p. 1. Other sources emphasized interconnectivity between individuals, groups of people, or between services and organizations, either in a theoretical model or through an intervention [46,50,51,53,55,56]. In this regard, Aldwin and Igarashi [55] propose that the collective efficacy of a community can increase the adaptive capacity of individuals. Therefore, it is recommended that initiatives should focus on including families, neighborhoods, and an umbrella support system involving a collaborative environment between various entities, such as governmental or non-governmental organizations and formal and informal stakeholders. Such services can address the unmet needs of stakeholders, better understand an individual’s experience, and, overall, promote community participation [46,50,51,53,56]. However, it is significant that the integration of leisure, commerce, and the business communities is not evident as part of the general ecosystem approach to improve the health and wellbeing of older people through community participation.

### 3.2. Ecosystem Mechanisms: What Works Well and What Prevents Effective Working?

The creation and maintenance of an age-friendly ecosystem for the community participation of older people depends on an existing and identified need, authorization, knowledge, planning, preparation, design, and virtual and/or place-based resources and attributes.

#### 3.2.1. Existing and Identified Need

The analysis indicates that there is an existing and identified need to provide support for older people. Needs can arise in relation to a critical event such as the COVID-19 pandemic. Lak et al. [49] suggest that to promote active aging, an ecological approach is needed, which addresses various types of needs such as social (e.g., social contact, networks, neighborliness), civic, financial (e.g., affordable housing), cultural (e.g., events, activities), and spiritual or religious. Bettis et al. [56] suggest that social needs can be met through relationships with family and friends, whereas mental health support can be provided through counseling. When considering ecosystem factors associated with successful aging, Jang [51] identifies the psychological need for emotional support and ways to heighten, reinforce, and build older adults’ self-esteem. Therefore, addressing such needs can enhance older people’s wellbeing and longevity, though it is important to consider that needs may vary from person to person [49].

#### 3.2.2. Authorization, Knowledge, Planning, Preparation, and Design

Forms of authorization required to create and maintain an ecosystem reside at the political, organizational, and personal level. Loos et al. [52] discussed the role of political and social movements such as the WHO Age-Friendly Cities and Communities (AFCC) initiative and the UN Sustainable Development Goals in legitimizing the notion of ecosystem developments for older people, whilst DeLaTorre and Neal [57] identified the importance of governmental support and collaboration in this respect. At the organizational level, Fulmer et al. [35] recognize ongoing age-friendly efforts such as certified age-friendly employers, whereas at a personal level, Wetle [36] notes that community champions are acknowledged as mechanisms through which ecosystems can be created. Baldissera et al. [46] suggest that this involves generating knowledge through examining organizations, attending to service structures/models, strategies, and solutions, and understanding the care needs of specific populations. Camarinha-Matos et al. [53] emphasize the importance of defining what an ecosystem should consist of and identifying the necessary supports to make it work and sustainable. Additionally, DeLaTorre and Neal [57] propose the use of action plans and committees as mechanisms to create ecosystems.

#### 3.2.3. Virtual and/or Place-Based Resources and Attributes

Some of the reviewed sources emphasized the importance of having place-based resources and attributes, such as open spaces, cleanliness, and safety, that are available, accessible, and in proximity to older adults [49,51]. In addition to physical resources, virtual or technological resources also play a crucial role in promoting community participation [54]. Camarinha-Matos et al. [53] developed an ambient assisted living framework that uses digital systems and information and communication technologies (ICT) support infrastructures to bring together various care services. Carroll et al. [50] aimed to unify community healthcare through online-based technological services, whereas Ferreira et al. [47] illustrated the need to go beyond telecentres to achieve the goal of fostering the digital inclusion of older people in Brazil. Moreover, two sources built upon the WHO Age-Friendly City initiative, using technology and ICT to enhance community participation and engagement [52,54]. Overall, these studies underscore the potential benefits of leveraging technology and digital resources to create age-friendly and inclusive communities.

### 3.3. Barriers to Ecosystem Success

When analyzing the success of ecosystems at the micro or individual level, one of the key barriers that older adults face, as identified in the selected studies, is limited knowledge, which can hinder their ability to access and use potential supports effectively [54]. At the meso or interactional level, family and neighborhood barriers were also said also play a significant role. These barriers include family’s financial constraints, partner’s health problems, unrealistic expectations from friends and family, and lower social and economic status [49]. Thus, health and economic environments can impact how older people access services within their communities. At the macro or broader organizational level, there were three key barriers to successful ecosystem development. Firstly, there is a lack of political commitment at the leadership and policy levels, which can hinder progress [35,36]. However, addressing social, community, and societal issues as priorities was suggested to increase political commitment [36]. Secondly, time and resources can be a challenge for policy development, implementation, and research, which can hinder the creation and maintenance of the ecosystem [35,49,50,57]. Limited resources to create new community hubs without segregating older people were also deemed a barrier to sustainable community participation [49,54]. Lastly, accessibility, particularly digital accessibility, is considered a significant barrier; for instance, low levels of internet access in Brazil can inhibit access to social and civic engagements [47].

### 3.4. Facilitators of Ecosystem Success

At the micro or individual level of analysis, the reviewed sources underscore the importance of ‘personal motivators’ as critical facilitators of ecosystem success. Ferreira et al. [47] highlight that personal motivators such as leisure, hobbies, and entertainment can serve as powerful catalysts for older adults to actively engage in ecosystem activities. Similarly, Jang [51] notes that older adults’ perceived control over their health and overall wellbeing can empower them to leverage ecosystem resources and participate actively in community life. Lak et al. [49] further emphasize the significance of older adults’ ability to live independently in the community, which motivates them to engage fully in ecosystem activities and maintain their health, according to their own objectives, capabilities, and opportunities.

At the meso or interactional level, facilitating mechanisms such as social capital, elimination of system silos, and equity and diversity play a crucial role in promoting successful ecosystems. Social capital, which encompasses norms of reciprocity, trust, social interactions, and civic participation, is essential for increasing active aging in community settings [49,57]. Furthermore, a strong and supportive social network can enhance the wellbeing and longevity of older individuals in society [49]. Community champions, who are integral components of social capital at the community level, are also identified as vital for maintaining and advancing the ecosystem [35,36].

Eliminating system silos is another critical component for developing successful ecosystems. Fulmer et al. [35] argue that it is essential to eliminate silos and ensure continuity across the care continuum. To achieve this goal, ecosystem stakeholders must coordinate across various sectors, all with the common purpose of creating age-friendly communities. By cultivating a strong sense of social connectedness and mutual support, stakeholders can effectively break down barriers between different areas of the ecosystem and create a more cohesive and effective system that better meets the needs of older adults [35].

In terms of equity and diversity, it is crucial to consider the role of broader age-friendly organizational coalitions when seeking to promote community participation for diverse groups of older people, as argued by Menec [48]. By prioritizing equity and diversity in ecosystem development, stakeholders can ensure that all individuals, regardless of their background or ability, feel welcome and included and able to develop a sense of community, which is essential for promoting the wellbeing and active participation of older adults.

At a macro level, there are several key factors that have been identified as important facilitators of successful ecosystems. These include policy and political facilitators, support systems, and the use of guiding frameworks. Policy and political facilitators involve political commitments towards ecosystem agendas [50], collaborative and holistic approaches to service provisions, unifying of digital and non-digital organizations, and ensuring continuity across the care continuum [35,36,50,51,53,57]. DeLaTorre and Neal [57] note that the interrelation of policies is crucial in creating the connective tissue of neighborhoods, upon which social connectivity is built. Additionally, support systems play a crucial role in facilitating successful ecosystems. These may include trained counselors [56], stakeholder innovation and involvement [50], involvement of international and national agencies (e.g., WHO) and government [48], and the involvement of academic researchers to ensure effective identification of needs and assessment of outcomes [36].

The use of guiding frameworks is also a crucial factor in the success of ecosystems. Guiding frameworks such as CASE or Ecological System Theory (EST) can provide a structured approach to ecosystem development and help to ensure its smooth functioning [36,48]. These frameworks can be adapted to suit the unique social, economic, and cultural context of a community and can be built on existing models developed by organizations such as the WHO [48]. By utilizing these frameworks, ecosystem stakeholders can identify key components and relationships within the ecosystem, facilitate collaboration and information sharing, and develop a comprehensive understanding of the needs of the community.

Overall, the reviewed sources highlight the importance of addressing these facilitators at the micro, meso, and macro levels to create successful ecosystems that promote active aging and enhance the wellbeing of older adults in the community.

### 3.5. Outcomes

While there may not have been specific evaluations of ecosystems in terms of their outcomes in facilitating the community participation of older people, several ‘outcomes’ were identified in relation to each of the different definitions of ‘ecosystem’. The concept of ecosystem has contributed to the development of various models and approaches that have had positive impacts on the aging population. These include the following:

- A community engagement program aimed at promoting healthy relationships and resilience as well as facilitating digital engagement. Specifically, the development of telecentres as a part of the ecosystem was found to be useful for improving digital engagement. This approach recognizes the importance of social connectedness and access to technology for promoting community participation among older people. However, it was noted that a broader multidimensional approach involving other ecosystem levels would be needed to fully promote digital inclusion [47].
- Active aging across the life-course [49]. This approach recognizes the potential of older people to contribute to their communities and society as a whole and aims to create environments that support their continued participation and inclusion (through lifelong learning, engagement in meaningful activities, and social connectedness).
- A range of key factors to assess ‘successful aging’ among older people aging-in-place, which are organized according to individual, family, and community systems (Jang). These factors may include individual characteristics such as physical and cognitive function, mental health, and social engagement; family-related factors such as social support, caregiving, and intergenerational relationships; and community-related factors such as access to healthcare and social services, neighborhood safety, and social and cultural opportunities.
- Applying ecological principles can facilitate the development of age-friendly communities, as found by [48]. According to DelaTorre and Neal [57], ensuring that cities maintain age-friendly policies requires ongoing planning initiatives that consider macro-level factors. When an ecological approach is used to develop age-friendly cities, as noted by Marston et al. [54], there is evidence of increased stability in areas such as education, support, and employment for older people.
- Politically, adopting an ecological perspective can facilitate political commitment and long-term policy planning towards creating age-friendly communities [35,36,50,57].
- At the policy level, changes that encourage the development of social and built environments promoting belonging and social engagement throughout the life course can facilitate the development of social capital, impacting both community and individual health and wellbeing [47,48,49,50,55,57]. This requires a focus on creating age-friendly environments that support community participation and social connectedness, including access to social and cultural activities, transportation, and public spaces that promote interaction and inclusivity.

### 3.6. Stakeholder Event

A stakeholder consultation event titled “Supporting Community Participation for Older People: Thinking About Inclusive Togetherness” was conducted on 1 July 2021, attended by 28 participants representing diverse academics, community, health and social care practitioners, and policymakers. The objective of the event was to present the study aims and discuss the analysis in relation to policy and practice issues. The following are the key messages that emerged from the discussion:

1. There exist several community-driven projects and initiatives aimed at building community resilience, which have not been documented in academic literature. The COVID-19 pandemic has further fueled the development of such initiatives, raising discussions on equity, diversity, inclusion, community responsibility, and local democracy. To create places that function across diverse older people and attract intergenerational participation, it is essential to avoid treating older people as a homogeneous group. This requires a focus on empowerment, especially amplifying the voices of those who are often overlooked. However, disempowerment over the years has made it challenging to sustain community-level change, and stronger policy commitment is required to foster community empowerment.
2. To avoid tokenistic participation, it is necessary to develop inclusive, intersectional, and cross-sectional ways of working that give more control and assets to the community. This requires collaboration between professionals, practitioners, and residents to ensure that everyone’s voices are heard and considered.
3. Initiating a debate around the concept of caring cities and communities would be useful to challenge organizational agendas and shift towards perspectives that prioritize the needs and preferences of both the city and its citizens.
4. Consider reframing the perception of older people as a homogenous group of frail individuals, as many of them are active community participants. By doing so, we can avoid the development of age silos and focus on community development for active aging. This will help in creating a better society for all ages and bridging the gap between young and old. Additionally, we need to counter the negative narrative of older people as a financial burden on society and instead highlight them as valuable resources and assets.
5. To move forward, community hubs and people’s assemblies are potential solutions, but these require a new community-based narrative that includes communities of interest and regional variations. This new way of thinking also requires political and community commitment as well as funding for social movements and networks—rather than one-off interventions, sustained efforts are necessary.
6. ‘Inclusive coffee mornings’ organized by a local church are an example of best practice for supporting older adults’ community participation. The initiative works thanks to its bottom-up approach, with older people helping each other, and its ability to also bring together diverse groups. However, initiating, and sustaining citizen-led initiatives can be challenging; they require dedication, time, and effort from volunteers and organizers alike.
7. A common pitfall of many policies is to segregate individuals based on their age groups, rather than recognizing that we are all unique individuals with our own personalities, stories, and experiences. A notable example of best practice is the V&A Dundee’s (the first design museum in Scotland) ‘See Me, Hear My Voice’ initiative, which forms part of the Dundee International Year of Older People. The initiative aims to move beyond viewing older people solely through demographic lenses and instead focuses on recognizing them as individuals with diverse backgrounds, interests, and skills—shifting from a ‘care’ perspective to a ‘community’ perspective, ultimately promoting the idea of caring communities.
8. Solutions for enhancing older people’s community participation need to be locally driven. Smaller communities are often better positioned to create innovative and effective approaches that work for their unique needs and circumstances. For instance, in Kirriemuir (a town located in the county of Angus, Scotland), collaboration with the Royal Town Planning Institute has led to the implementation of new traffic calming measures, road crossings, signage, and community garden spaces, which benefit people of all ages in the community. However, it is essential to acknowledge that what works in one area may not necessarily work in another. Therefore, it is crucial to learn from each other by sharing stories and experiences. This is where community champions can play a vital role in facilitating the development of new initiatives and the sharing of knowledge.

These insights have informed the development of a set of recommendations which are presented in the Discussion section below.

## 4. Discussion and Conclusions

This review investigated the effectiveness of age-friendly ecosystems in promoting community participation among older adults through an analysis of their contexts, mechanisms, and outcomes. The study highlighted that age-friendly ecosystems are context-dependent, meaning that the success of these ecosystems can vary depending on the context in which they exist, are implemented, and how they are conceptualized. Across most of the reviewed sources, the term ‘ecosystem’ was used to describe a complex system of multiple actors, organizations, and environments, which included older adults themselves, healthcare providers, community champions, formal and informal carers, along with voluntary and community sectors, as stakeholders within the ecosystem. This approach recognizes the importance of stakeholders working together and considers the multiple environments in which older adults live to help consider individuals’ unique needs and circumstances.

The majority of the sources included in this review suggest that ecosystems can be conceptualized as models or frameworks, with several sources [35,36,47,48,51,55,57] drawing on Lawton and Nahemow’s ‘ecological theory of aging’ [58] or Bronfenbrenner’s [42] ‘ecological systems’ theoretical framework. As mentioned in the methodology section, Bronfenbrenner’s ecological systems framework provided the theoretical framework for the present realist review’s data extraction and analysis. The realist review approach sought to identify underlying mechanisms and contextual factors that contribute to the effectiveness of interventions, and the application of an adapted version of Bronfenbrenner’s model allowed for a comprehensive analysis of the interaction and impact of various environmental factors and their influence on the outcomes of age-friendly related interventions.

All reviewed studies, except for one [55], generally grouped ‘older adults’ or ‘older people’ as a homogenous population, solely defined by their age and considered to be disadvantaged. However, it is essential to recognize and address the diversity that exists among older adults in terms of age, gender, ethnicity, and other factors within the broader context of the ecosystem framework. Failing to recognize this diversity can result in inequitable outcomes and leave some groups with unmet needs. Therefore, it is crucial to ensure that age-friendly interventions are designed to promote equity and consider the unique perspectives and requirements of all older adults. This can be achieved with a holistic socio-technical approach to ecosystem design and by incorporating a more inclusive and nuanced understanding of the older adult population, which recognizes and values diversity, and by implementing strategies that address the specific needs and challenges faced by different groups of older adults [10,11,49,51].

By implementing an age-friendly ecosystem that addresses the unique needs of older people, we can promote their social, emotional, and physical wellbeing, and enhance their quality of life. Such an ecosystem will foster an environment of mutual respect, inclusion, and support, thereby ensuring that older individuals can continue to contribute to their communities in meaningful ways. Ultimately, the creation and maintenance of an age-friendly ecosystem will benefit society by enabling older individuals to lead fulfilling and rewarding lives.

According to the review results, there are key facilitating mechanisms that underlie successful age-friendly ecosystems, including the availability of physical and social infrastructure, opportunities for social engagement, and the provision of accessible and affordable services. This includes access to healthcare, social services, community resources as well as virtual or technological resources that are tailored to the needs and preferences of older adults [36,47,48,49,50,55,57]. Age-friendly ecosystems can also help promote healthy behaviors, such as physical activity, by providing access to resources and opportunities that support these behaviors. In line with research evidence on age-friendly communities [10,11,13,26,30,61], these findings reinforce the importance of designing age-friendly ecosystems that are responsive to the needs and preferences of older adults. Furthermore, it is evident that age-friendly ecosystems can improve community participation among older adults when they are designed to be inclusive, participatory, and collaborative. By doing so, communities can enhance the quality of life for older adults, promote social connections and community engagement, and create more vibrant, sustainable communities for all residents.

The review highlights several facilitators of success in developing ecosystems that promote community participation among older adults. At the individual level, personal motivators (e.g., leisure, hobbies, entertainment), perceived control over health and wellbeing, and the ability to live independently in the community motivate older adults to actively engage in community activities [47,49,51]. At the meso level, the review identified three relevant facilitators of ecosystem success: cultivation of social capital (e.g., shared values, trust, social norms, and mutual support) through civic participation; elimination of system silos; and the promotion of equity and diversity [35,36,49,57]. By deliberately considering and implementing these facilitators, ecosystem stakeholders can foster a sense of belonging and connectedness, which is essential for creating thriving ecosystems and promoting the wellbeing and active participation of older adults in society.

At the macro level, the success of ecosystems is facilitated by various factors, including policy and political commitments towards ecosystem agendas, supportive systems, and the use of guiding frameworks, such as the WHO Age-Friendly Cities Framework, which help ensure a structured approach to ecosystem development [35,36,48,50,51,53,56,57]. Arguably, it is important to prioritize these factors at the macro level to ensure that initiatives are sustainable and impactful, as this approach can lead to more effective resource allocation and service provision, ultimately resulting in better outcomes for those involved in the ecosystem.

The review also indicates that older adults face multiple barriers to community participation at various levels of the ecosystem. Limited knowledge hinders their ability to access and use potential supports, while family and neighborhood barriers, such as financial constraints and lower social and economic status, can impact their access to services [49,54]. Lack of political commitment at leadership levels, limited resources, and poor accessibility, particularly digital accessibility, are significant barriers at the macro level [35,36,47,49,50,54,57]. Recognizing and addressing these barriers is crucial for successful ecosystem development and creating age-friendly communities that promote equity and inclusivity for all older adults. This means that policymakers and community leaders must take proactive measures to understand and address the factors that limit older adults’ participation in their communities. This may require a more significant commitment to developing policies and programs that prioritize the needs and perspectives of older adults, as well as ensuring that these programs are adequately resourced and accessible to all.

Accordingly, the consultation event on supporting community participation for older people highlighted the importance of community-based approaches and avoiding framing older people as a homogeneous group. Grassroots projects and initiatives in local communities should be supported, with an emphasis on empowerment and the voices of those who are seldom heard. To better support older adults, more inclusive ways of working between professionals, practitioners, and residents are necessary. It is essential to avoid age silos, as they may lead to the stigmatization and marginalization of older adults. Community hubs and people’s assemblies are a way forward; however, new community-based narratives and social movements are necessary to fully realize the potential of these approaches. Examples of good practice include citizen-led initiatives, risk-enabling approaches, and local solutions tailored to the specific needs and strengths of the community. Finally, there is a need to share stories and experiences to learn from each other and foster the development of community champions who can drive forward local initiatives.

The added value of this review lies in the development of a set of recommendations for promoting healthy and active aging. These recommendations offer valuable insights into the mechanisms and contextual factors that contribute to the success of age-friendly ecosystems, with a focus on creating multi-layered environments that address the needs of diverse individuals and communities, including older people. The recommendations highlight the importance of an interplay between individual, social, and physical components alongside policymaking processes to promote increased physical activity and better health outcomes. Collaborative working among diverse stakeholders at the community level is also recommended to identify needs, develop inclusive ecosystems, and assess outcomes. Additionally, the recommendations emphasize the significance of intergenerational relationships, equity, diversity, and political commitment to support sustainable communities (see Table 3 for detailed recommendations).

Strengths and Limitations: One of the strengths of this rapid realist review is that it provides a comprehensive examination of the factors that contribute to the success of age-friendly ecosystems that are inclusive, collaborative, intersectional, and responsive to the needs and preferences of older adults, while promoting community participation. This review process is guided by a clear program theory (i.e., Bronfenbrenner’s adapted ‘ecological systems framework’)—lack of theory or a tightly constrained micro theory might have limited the relevance or usefulness of the findings. The review also highlights that age-friendly ecosystems need to be designed with older adults’ needs and preferences in mind and that their success depends on the availability of physical and social infrastructure, opportunities for social engagement, and accessible and affordable services.

However, some potential limitations were also identified. Most of the studies reviewed were conducted in the USA, which could reflect bias in favor of US-based studies within the databases searched. This may be attributed to the inclusion criteria, which only included English-language studies. Despite these limitations, this review offers an international perspective on the current evidence for age-friendly ecosystems. Notably, the literature on this area is still emerging, and there is limited empirical evidence to support the effectiveness of these ecosystems, particularly in terms of specific evaluations of their outcomes in facilitating the community participation of older people. Another challenge associated with this RRR is the potential trade-off between speed and rigor, as this type of review requires a balance between timeliness and methodological rigor [38]. While it is possible that some relevant publications may have been omitted from the study, the authors believe that the selected publications are sufficient to achieve the review’s aim, and any such omissions would not substantially affect the study’s findings.

Overall, a rapid realist review is a valuable approach for generating evidence in a timely and relevant manner and can be especially useful for decision-making in emerging areas of research, such as age-friendly ecosystems. Future studies should include measurement of outcomes to assess the effectiveness of ecosystem interventions for age-friendly places and communities which encourage the community participation of older people. Future research should also consider the heterogeneity amongst older people to ensure that age-friendly ecosystems address the needs and experiences of all older adults, including those from diverse backgrounds and with various levels of ability, or those from communities who are seldom heard. Such research could also help identify potential disparities in access to support programs and services and inform interventions to address them. In addition, further investigation is needed to explore the mechanisms and contextual factors that contribute to successful outcomes among older adults, particularly those who face social, economic, or health-related challenges. Going forward, research is required which prioritizes genuine and inclusive collaborative working among diverse stakeholders, including older people themselves, as this could enhance the relevance and effectiveness of interventions, and future studies should examine the impact of such collaboration on outcomes. Finally, research is needed on effective approaches and interventions for creating more age-friendly ecosystems in different contexts and communities, and especially on how these interventions can be sustained over time. Researchers, practitioners, policymakers, and other stakeholders working to promote healthy, active aging and community participation for older adults will find this study to be a valuable reference for gaining a comprehensive understanding into the key issues, challenges, and opportunities in this area, as well as some of the most effective approaches and interventions for creating more age-friendly ecosystems.

## Figures and Tables

**Table 1:** Example of search terms and a search string used in the Rapid Realist Review.

Concept	Search Terms
Older people	Old* people
	Old* adult
	Senior*
	Senior citizen
	Age*
	Elder*
	Over 55′s
Ecosystem	Ecosystem
	Socio-ecosystem
	Socio ecosystem
Participation	Participation
	Engagement
	Involvement
	Social participation
	Civic participation
	Inclusion
	Social capital
Community	Community
	Neighbourhood
	Neighborhood
	Place
Example Search String for Web of Science	

ALL = (Older people OR older adults OR senior citizen OR over 55 s OR aged OR elder*) AND ALL = (ecosystem OR socio-ecosystem OR socio ecosystem) AND ALL = (community participation OR engagement OR involvement OR social participation OR civic participation OR neighbourhood OR place OR inclusion OR social capital OR participation OR community).

**Table 2:** Characteristics of the studies included in the Rapid Realist Review.

Authors	Title	Publication Type	Publication Title	Publication Date	Main Area of Research	Main Author Location	Study Geographical Focus	Study Design
Aldwin, C. and Igarashi, H.	An ecological model of resilience in late life.	Journal article	Annual review of gerontology and geriatrics	2012	Aging	USA	Global	Review
Baldissera, Thais, and Camarinha-Matos, Luis M	SCoPE: Service Composition and PErsonalization Environment	Journal article	Applied Sciences	2018	Science and Technology	Brazil	Brazil and Portugal	Constructive researchmethod
Bettis, J., Kakkar, S. and Chan, C, D.	Taking Access to the Community: An Ecological Systems Framework for In-Home Counselling with Older Adults	Journal article	Adultspan Journal	2020	Mental health care/Counselling	USA	Global	Review
Camarinha-Matos, L, M., Rosas, J., Oliveira, A. I. and Ferrada, F.	Care services ecosystem for ambient assisted living,	Journal article	Enterprise Information Systems,	2015	Science and Technology	Portugal	Europe	Conceptual model
Carroll, N., Kennedy, C. and Richardson, I.	Challenges towards a Connected Community Healthcare Ecosystem (CCHE) for managing long-term conditions.	Journal article	Gerontechnology	2016	Science and Technology	Ireland	Europe, North America, and Australasia	Systematic mapping study
DeLaTorre, A. and Neal, M, B	Ecological Approaches to an AgeFriendly Portland and Multnomah County	Journal article	Journal of Housing for the Elderly	2017	Aging	USA	USA	Reflective account
Ferreira, S, M., Sayago, S. and Blat, J.	Going Beyond Telecenters to Foster the Digital Inclusion of Older People in Brazil: Lessons Learned from a Rapid Ethnographical Study	Journal article	Information Technology for Development	2016	Technology	Brazil	Brazil	Rapid ethnographic study
Fulmer, T., Patel, P., Levy, N., Mate, K., Berman, A., Pelton, L., Beard, J., Kalache, A., and Auerbach, J.	Moving Toward a Global Age-Friendly Ecosystem	Journal article	J Am Geriatr Soc	2020	Aging	USA	Global	Retrospective account of the global progress made toward age-friendly ecosystem
Jang, H. Y.	Factors associated with successful aging among community-dwelling older adults based on ecological system model	Journal article	International Journal of Environmental Research and Public Health	2020	Nursing	Korea	Korea	Descriptive secondary analysis
Lak, A., Rashidghalam, P., Myint, P. K., and Baradaran, H. R.	Comprehensive 5P framework for active aging using the ecological approach: an iterative systematic review	Journal article	BMC Public Health	2020	Architecture and Urban Planning	Iran	Global	Narrative review
Loos, E., Soubati, M., and Behrendt, F.	The Role of Mobility Digital Ecosystems for Age-Friendly Urban Public Transport: A Narrative Literature Review	Journal article	International Journal of Environmental Research and Public Health	2020	Digital health information	Netherlands	Global	Narrative literature review
Marston, H.R., Shore, L., and White, P.J.	How does a (Smart) Age-Friendly Ecosystem Look in a Post-Pandemic Society?	Journal article	International Journal of Environmental Research and Public Health	2020	Digital technology, Wellbeing and Social Care	UK	Global	Review
Menec, V.H.	Conceptualizing Social Connectivity in the Context of Age-Friendly Communities.	Journal article	Journal of Housing for the Elderly	2017	Aging, Community health	Canada	Global	Review, Conceptual model
Wetle, T.T.	Age-Friendly Ecosystems: An Aspirational Goal	Editorial	Journal of the American Geriatrics Society	2020	Public Health	USA	Global	Editorial comment on another published article

**Table 3:** Recommendations for the success of age-friendly ecosystems.

Adopt a multi-layered ecosystem approach that integrates tailored interventions targeting individual, social, and physical components alongside policy-making processes to enable healthy and active aging. This approach should focus on developing environments that promote increased physical activity and better health outcomes. Conduct a community needs assessment of older people from intersectional and intergenerational perspectives to address diverse needs and interests. Ensure that all elements of the ecosystem interconnect and function harmoniously to improve healthy and active aging for diverse people of different ages, cultures, religions, and backgrounds. This will require an interplay between the physical and virtual environment and individuals in all aspects of community building, including planning, transport, support services, business/commerce, and leisure. Promote collaborative working among diverse stakeholders at the community level, including health and social care practitioners, businesses, the retail and commercial sectors, educators, academics, international and national agencies (e.g., WHO, national and local government), and residents to identify needs, develop inclusive ecosystems, and assess outcomes. This will ensure political will and support for community-level engagement. Ensure that place-making for and with older people is fully considered in the design, functionality, and experience of the ecosystem. Attention to the development of community hubs that embrace intergenerational relationships through education, leisure, and access to services is key to a thriving ecosystem. Go beyond a one-size-fits-all approach and instead focus on equity and diversity. It is important to recognize that older adults are a heterogeneous group with diverse needs, interests, and backgrounds. Gain political commitment at leadership and policy levels to enable the creation and maintenance of community-based ecosystems with place-based resources and attributes to support sustainable communities.

## Data Availability

The data that support the findings of this study are available on request from the corresponding author, J.S.

## Supplementary Materials

The following supporting information can be downloaded at: https://www.mdpi.com/article/10.3390/geriatrics8030052/s1, Table S1: PRISMA Checklist; Figure S1: PRISMA flow diagram; Figure S2: Updated PRISMA flow diagram [62].
