# Supplementary material for: Enhancing Community Participation through Age-Friendly Ecosystems: A Rapid Realist Review
Source: Geriatrics (Basel). 2023 May 11;8(3):52. doi: 10.3390/geriatrics8030052 (PMC10204480; doi:10.3390/geriatrics8030052)
Supplement: Supplementary file 1 [file geriatrics-08-00052-s001.zip › Figure S2 PRISMA_flow_diagram_updated.pdf]

Figure S2: Updated PRISMA flow diagram

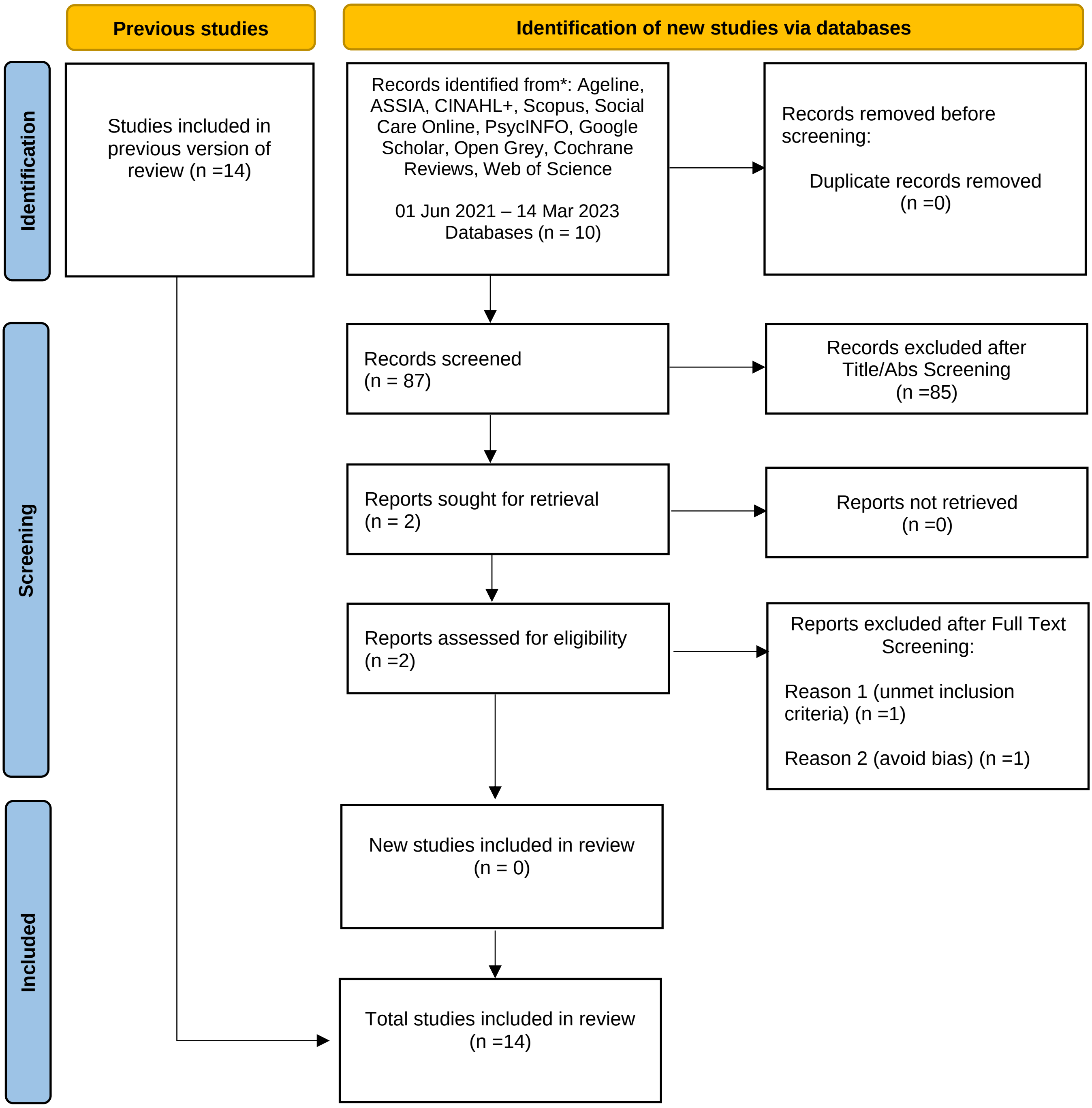

Based on Page MJ, McKenzie JE, Bossuyt PM, Boutron I, Hoffmann TC, Mulrow CD, et al. The PRISMA 2020 statement: an updated guideline for reporting systematic reviews. BMJ 2021;372:n71. DOI: 10.1136/bmj.n71
